# Interactions of Sodium Salicylate with β-Cyclodextrin and an Anionic Resorcin[4]arene: Mutual Diffusion Coefficients and Computational Study

**DOI:** 10.3390/ijms24043921

**Published:** 2023-02-15

**Authors:** Diana M. Galindres, Nicolás Espitia-Galindo, Artur J. M. Valente, Sara P. C. Sofio, M. Melia Rodrigo, Ana M. T. D. P. V. Cabral, Miguel A. Esteso, Jhon Zapata-Rivera, Edgar F. Vargas, Ana C. F. Ribeiro

**Affiliations:** 1 Grupo de Fisicoquímica y Análisis Matemático (Physchemath), Facultad de Ciencias y Humanidades, Universidad de América, Avda Circunvalar No. 20-53, Bogotá 110321, Colombia; 2 Grupo de Termodinámica de Soluciones, Departamento de Química, Facultad de Ciencias, Universidad de los Andes, Cra 1 No. 18A-12, Bogotá 111711, Colombia; 3 Molecular Electronic Structure Group, Department of Chemistry, Universidad de los Andes, Carrera 1 No. 18A-10, Bogotá 111711, Colombia; 4 CQC-IMS, Department of Chemistry, University of Coimbra, 3004-535 Coimbra, Portugal; 5 U.D. Química Física, Universidad de Alcalá, 28805 Alcalá de Henares, Spain; 6 Faculty of Pharmacy, University of Coimbra, 3000-548 Coimbra, Portugal; 7 Faculty of Health Sciences, Universidad Católica de Ávila, Calle Los Canteros s/n, 05005 Ávila, Spain

**Keywords:** β-cyclodextrin, sodium sulfonated resorcinarenes, sodium salicylate, diffusion by Taylor technique, transport properties, computational calculations

## Abstract

The interaction between sodium salicylate (NaSal) and the two macrocycles 5,11,17,23-tetrakissulfonatomethylene-2,8,14,20-tetra(ethyl)resorcinarene (Na_4_EtRA) and β-cyclodextrin (β-CD) has been studied by the determination of ternary mutual diffusion coefficients, and spectroscopic and computational techniques. The results obtained by the Job method suggest that the complex formation is given in a 1:1 ratio for all systems. The mutual diffusion coefficients and the computational experiments have shown that the β-CD-NaSal system presents an inclusion process, whereas the Na_4_EtRA-NaSal system forms an outer-side complex. This fact is also in line with the results obtained from the computational experiments, where the calculated solvation free energy has been found to be more negative for the Na_4_EtRA-NaSal complex because of the partial entry of the drug inside the Na_4_EtRA cavity.

## 1. Introduction

β-Cyclodextrins (β-CD) are macrocycles (cyclic oligosaccharides) made up of ring-shaped monosaccharide molecules, which are produced from starch by enzymatic conversion [1]. β-CD are non-toxic, edible, non-hygroscopic, chemically stable, and easily separable macrocycles. As a consequence, among the main uses that have been found for them are for the food, pharmaceutical, and drug delivery systems, and in industries such as chemical, agricultural, and environmental [1,2]. Its use in the pharmaceutical field is due to their ability to improve drug solubility and stability [3]. In addition, they have the ability to form supramolecular complexes with various chemical compounds of biological and pharmacological interest [1,2,3,4,5].

Other macrocycles of great importance in supramolecular chemistry are Resorcinarenes (RA) [6]. These compounds can be functionalized on the lower rim with hydrocarbon chains, which allows for the hydrophobicity modification of the macrocycle. Different functional groups can be added to the upper rim, such as the sulfonate group, which increases their solubility in water and allows the interaction of these compounds with solutes of pharmaceutical interest. RAs present a cavity that, under specific conditions, can be used for the inclusion of different guests by the formation of host–guest complexes (HG) [7].

Sodium salicylate (NaSal) is a compound of great interest due to its anti-inflammatory, analgesic, and antipyretic properties [8]. Additionally, it has been found that this compound induces apoptosis in cancer cells and also necrosis [8]. Due to its size and importance at pharmacological level, the study of its complexation is of great interest. Some earlier studies on the complexation of NaSal with β-CD are collected in the literature [9,10], although with results inconsistent with each other. Indeed, Junquera et al. [9], based on speed of sound and conductivity experiments, justify the presence of an NaSal-β-CD inclusion complex, according to what is usually found for most drug-CD complexes. Conversely, Deosarkar et al. [10] deduce that the external–lateral complex must be dominating over the inclusion complex. They base this conclusion mainly on quantum mechanical calculations using the DFT (Density Functional Theory) and by considering three conformers of β-CD with different patterns of hydrogen bonding between the primary hydroxyls and the salicylate (one of them with a bucket-like structure, conformer A, from which they obtain that the inclusion complex is the stable form; and the other two, conformers B and C, with a barrel-like structures, from which they deduce that the formation of the outer-side complex is the most favored). This type of discrepancy makes it convenient to further study the structure of the NaSal-β-CD complex. Likewise, the study of the interactions that may occur between NaSal and other macrocycles such as sodium 5,11,17,23-tetrakissulfonatomethylene-2,8,14,20-tetra(ethyl)resorcinarene (Na_4_EtRA) and its comparison with those that take place in the case of the NaSal-β-CD complex becomes of great interest.

Knowledge of the structure of the macrocycle-NaSal complex is of great importance. It allows for knowing if the NaSal is totally or partially encapsulated inside the macrocycle or how the NaSal is oriented inside the macrocycle cavity, offering greater protection and greater possibilities of being applied by using different delivery transit routes towards the therapeutic target, as well as greater efficiency in drug delivery and dosage [11] or, conversely, if an outer-side complex is formed, in which case the therapeutic transit routes of this macrocycle–NaSal formulation are more limited.

In this work, the diffusion and complexation of β-CD-NaSal and Na_4_EtRA-NaSal adducts is studied by using experimental (Taylor dispersion, UV–Vis, and NMR spectroscopy) techniques. In addition, both the molecular and the electronic structure of these complexes is studied by using computational techniques. The results found indicate that in both cases, host–guest type supramolecular complexes are formed between the macrocycle (Na_4_EtRA or β-CD) and NaSal, which enables the use of these results in drug delivery systems.

## 2. Results and Discussion

### 2.1. Ternary Diffusion Coefficients NaSal (Component 1) + Na_4_EtRA (Component 2)

Table 1 shows our data of *D*_11_, *D*_12_, *D*_21,_ and *D*_22_. They are the mean values of between four and six replicates measured for each concentration of NaSal (1) and Na_4_EtRA (2). As it can be ascertained, the coefficients *D*_11_ and *D*_22_ present standard deviation values generally lower than (±0.015 × 10^−9^ m^2^ s^−1^), while the cross-coefficients present higher values (±0.025 × 10^−9^ m^2^ s^−1^).

From this table, it can be seen that while *D*_11_ increases, *D*_22_ decreases with the solute fraction of NaSal (*X*_1_ = *C*_1_/(*C*_1_ + *C*_2_)). At the limiting situations of *X*_1_ = 0 and *X*_1_ = 1, the values of *D*_11_ correspond, respectively, to the tracer diffusion coefficient of NaSal in Na_4_EtRA solutions (*D*_11_ = 0.979 × 10^−9^ m^2^ s^−1^) and the binary mutual diffusion coefficient of aqueous NaSal (*D*_11_ = 1.096 × 10^−9^ m^2^ s^−1^). Regarding this last value found here for *D*_11_, a good agreement is observed between it and the binary diffusion coefficient value already obtained in previous works (*D* = 1.020 ± 0.009) [12].

Similarity, *D*_22_ values at the limiting situations of *X*_1_ = 0 and *X*_1_ = 1 are the binary mutual diffusion coefficient of aqueous Na_4_EtRA (*D*_22_ = 0.711 × 10^−9^ m^2^ s^−1^) and the tracer diffusion coefficient of Na_4_EtRA in aqueous NaSal solutions (*D*_22_ = 0.499 × 10^−9^ m^2^ s^−1^), respectively. In this case, the excellent agreement between the first limiting value and the value of the binary diffusion coefficient obtained for aqueous solutions of Na_4_ETRA at the same concentration (that is, differences around 1.5%) can also be verified [13,14].

In relation to the behavior of the cross-diffusion coefficients, *D*_12_ and *D*_21_, it is observed that these are all positive, indicating that a co-current coupled flow exists. However, it is observed that *D*_21_ is practically zero, within the uncertainty of the method (<3%). On the other hand, as the fraction of the solute NaSal increases, *D*_12_ also increases, reaching its maximum value for *X*_1_ →1, illustrating the existence of significant co-current coupled flows. From the values of the *D*_12_/*D*_22_ ratio, it can be seen that one mole of diffusing Na_4_EtRA co-transports at most 2.4 mol of NaSal, whereas the values of the *D*_21_/*D*_11_ ratio show that one mole of diffusing NaSal can co-transport up to 0.10 mol of Na_4_EtRA. At the other limit *X*_1_ →0, *D*_12_ is zero since it is not possible for the Na_4_EtRA concentration gradients to produce coupled flows of NaSal in solutions in which NaSal is absent.

**Figure 1:**
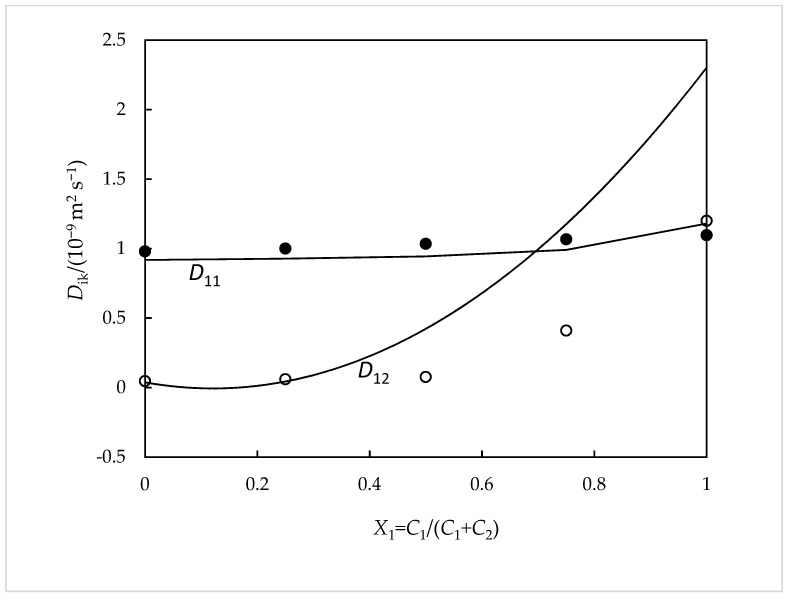
Ternary *D_ik_* coefficients for aqueous NaSal(*C*_1_) + Na_4_EtRA(*C*_2_) solutions containing 0.010 mol dm^−3^ of total solute and their comparison with the predicted *D_ik_* coefficients calculated from the Nernst equations: *D*_11_, filled circles; *D*_12_, hollow circles. The solid curves represent Nernst predictions [15].

**Figure 2:**
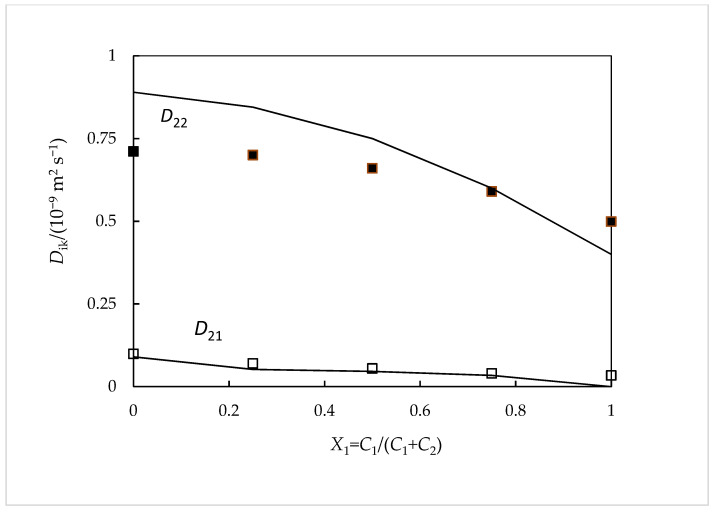
Ternary *D_ik_* coefficients for aqueous NaSal(*C*_1_) + Na_4_EtRA(*C*_2_) solutions containing 0.010 mol dm^−3^ of total solute and their comparison with the predicted *D_ik_* coefficients calculated from the Nernst equations: *D*_21_, hollow squares; *D*_22_, filled squares. The solid curves represent Nernst predictions [15].

For a better understanding of our experimental data (Table 1), a comparison was carried out between them and those predicted from Nernst equations [15] (Figure 1 and Figure 2). These equations are very useful for the qualitative comprehension of the main characteristics of the results. In them, both the coupled diffusion mechanism and the composition dependence of the *D_ik_* coefficients are included. This theory is well described in previous works [16]. The diffusion coefficients of the ionic species Na^+^, Sal^−^, and EtRA^4−^$(D_{i}^{0}$) were evaluated by using Equation (3), representing *F*, R, *T*, and $\lambda_{i}^{0}$ the Faraday constant, the gas constant, the temperature, and the limiting ionic conductivity.

(1) $$D_{i}^{0}=\frac{RT\lambda_{i}^{0}}{z_{i}^{2}F^{2}}$$

In the present work, the limiting ionic conductivity values for Na^+^, Sal^−^, and EtRA^4−^ ions and of the respective diffusion coefficients are $\lambda_{\mathrm{Na}+}^{0}$ = 50.1 × 10^−4^ S m^2^ s^−1^ [17], $\lambda_{\mathrm{Sal}-}^{0}$ = 34.5 × 10^−4^ S m^2^ s^−1^ [18] and $\lambda_{{\mathrm{EtRA}}^{4-}}^{0}$ = 229.3 × 10^−4^ S m^2^ s^−1^ [14], and $D_{{\mathrm{Na}}^{+}}^{0}$= 1.334 × 10^−9^ m^2^ s^−1^, $D_{{\mathrm{Sal}}^{-}}^{0}$ = 0.918 × 10^−9^ m^2^ s^−1^, and $D_{{\mathrm{EtRA}}^{4-}}^{0}$ = 0.384 × 10^−9^ m^2^ s^−1^.

From the analysis of these figures, it is possible to verify that our data are reasonably consistent with these theoretical values. It is worth highlighting the achievement of positive values for *D*_12_. A possible explanation for this fact can be given based on the different mobilities of the ions involved. The values of the mobility and the diffusion coefficient of the Na^+^ ions are higher than those of the RS^4−^ ions. Therefore, it must be concluded that the existence of a Na_4_EtRA concentration gradient causes the existence of an electric field that tends to slow down the Na+ ions and accelerate the RS^4−^ ions, since the diffusion path must always remain electrically neutral. If there is NaSal in the solution, the electric field generated by a concentration gradient of Na_4_RS will cause a coupled flow of Sal^−^ ions in the direction of the flow of RS^4−^ ions. This situation collaborates to explain the reasons for the positive values obtained for the *D*_12_ cross-coefficient. Consequently, it can be said that there are no favorable conditions for the formation of inclusion complexes between this drug and these macromolecules. Support for this fact is given by the results found from ^1^H NMR spectroscopy and computational studies which suggest that there is indeed no complexation by inclusion; however, the formation of outer-side complexes is not discarded.

### 2.2. Ternary Diffusion Coefficients NaSal (C_1_) + β-CD (C_2_)

Table 2 summarizes our data of *D*_11_, *D*_12_, *D*_21_, and *D*_22_. They are the mean values of between four and six replicates measured for each concentration of NaSal (1) and β-CD (2). As it can be ascertained, the coefficients *D*_11_ and *D*_22_ present standard deviation values generally lower than (±0.015 × 10^−9^ m^2^ s^−1^), while the cross-coefficients present higher values (±0.025 × 10^−9^ m^2^ s^−1^).

From this table, it can be verified that as the solute fraction of NaSal increases (*X*_1_ = *C*_1_/(*C*_1_ + *C*_2_)), *D*_11_ and *D*_22_ increase. In the limits *X*_1_ = 0 and *X*_1_ = 1, *D*_11_ represents the tracer diffusion coefficient of NaSal in 0.0100 mol dm^−3^ β-CD solutions, and the binary diffusion coefficient of NaSal in aqueous solutions at 0.0100 mol dm^−3^, respectively. This last value is in good agreement with the value found in the literature (3.4% deviation) [19], within the uncertainty of the method.

For the same composition limits, the *D*_22_ values represent the binary mutual diffusion coefficient of aqueous β-CD (i.e., *D* = 0.460 ± 0.020) and the tracer diffusion coefficient of β-CD in aqueous NaSal solutions (i.e., *D* = 0.470 ± 0.020), respectively.

Contrarily to the previous ternary system (NaSal + Na_4_EtRA), it is observed that for the present aqueous system (NaSal + β-CD), the *D*_12_ coefficient values are negative, reaching the maximum at *X*_1_ →1. This means that in this case there are significant counter-current coupled fluxes, resulting from the binding between the Sal^−^ anion and β-CD entities. On the other hand, *D*_21_ is practically zero for this range of concentrations, within the uncertainty of the method. From the *D*_12_/*D*_22_ ratio values, it can be seen that one mole of diffusing β-CD counter-transports at most 0.50 mol of NaSal, whereas the *D*_21_/*D*_11_ ratio values show that one mole of diffusing NaSal can counter-transport up to 0.04 mol of β-CD. These results show that the β-CD concentration gradients drive significant coupled flows of NaSal and, consequently, lead to favorable conditions for the formation of inclusion complexes. This effect is less pronounced when the influence of NaSal on the diffusion of β-CD is considered since the mobility of these species is expected to be quite similar.

To support these facts, the model developed by Paduano et al., whose description is well documented in the literature [20,21], can be applied in this case. Briefly, this model assumes the formation of a 1:1 supramolecular complex (Sal-β-CD), where the association constant *K*_a_ is given by the equilibrium between salicylate (Sal^−^) and β-cyclodextrin (β-CD) and the complex:

Sal^−^ + β-CD ↔ Sal-β-CD (2)

according to

(3) $$K_{a}=\frac{C_{\mathrm{Sal}-\beta -\mathrm{CD}}}{C_{{\mathrm{Sal}}^{-}}\times C_{\beta -\mathrm{CD}}}$$

where *C*_Sal_^−^ and *C*_β-CD_ are the concentrations of free (uncomplexed) Sal^−^ and β-CD, respectively, and *C*_Sal-β-CD_ is the concentration of the Sal-β-CD complex. These concentrations are correlated with each other by the following mass balance equations:

*C*_1_ = *C*_Sal_ + *C*_Sal-β-CD_ (4)

*C*_2_ = *C*_CD_ + *C*_Sal-β-CD_ (5)

Equations (6) to (9) provide the following relationships between the experimental mutual diffusion coefficients *D*_11_, *D*_12_, *D*_21_, and *D*_22_, measured for the total solute Sal^−^ (1) + β-CD (2), and the diffusion coefficients *D*_Sal_^−^, *D*_β-CD_, and *D*_Sal-β-CD_ which represent the diffusion coefficients of the free salicylate anion, the free β-CD and the complex, respectively, in solution. The values for these coefficients are shown in Table 3.

(6) $$D_{11}=D_{\mathrm{Sal}-}+\left(D_{\mathrm{Sal}-\beta -\mathrm{CD}}-D_{\mathrm{Sal}-}\right)\frac{\partial C_{\mathrm{Sal}-\beta -\mathrm{CD}}}{\partial C_{1}}$$

(7) $$D_{12}=\left(D_{\mathrm{Sal}-\beta -\mathrm{CD}}-D_{\mathrm{Sal}-}\right)\frac{\partial C_{\mathrm{Sal}-\beta -\mathrm{CD}}}{\partial C_{2}}$$

(8) $$D_{21}=\left(D_{\mathrm{Sal}-\beta -\mathrm{CD}}-D_{\beta -\mathrm{CD}}\right)\frac{\partial C_{\mathrm{Sal}-\beta -\mathrm{CD}}}{\partial C_{1}}$$

(9) $$D_{22}=D_{\beta -\mathrm{CD}}+\left(D_{\mathrm{Sal}-\beta -\mathrm{CD}}-D_{\mathrm{CD}}\right)\frac{\partial C_{\mathrm{Sal}-\beta -\mathrm{CD}}}{\partial C_{2}}$$

The diffusion coefficient value of free Sal anion (*D*_Sal_^−^ = 0.918 × 10^−9^ m^2^ s^−1^) is given in reference [19], while the diffusion coefficient value of free β-cyclodextrin molecules (*D*_β-CD_ = 0.436 × 10^−9^ m^2^ s^−1^) is estimated from *D*_22_ at *X*_1_ = 0. The diffusion coefficient of the Sal-β-CD complex (*D*_Sal-β-CD_ = 0.400 × 10^−9^ m^2^ s^−1^) is estimated from the Stokes–Einstein approximation (Equations (10) and (11)), which for a given species relates its diffusion coefficient with its effective radius, *r_h_*, and therefore to the cube root of its molecular volume:

(10) $$D=\frac{k_{B}T}{6\pi \eta r_{h}}$$

(11) $$D_{Sal-\beta -\mathrm{CD}}={\left(D_{\mathrm{Sal}-}^{-3}+D_{\beta -\mathrm{CD}}^{-3}\right)}^{-1/3}$$

*k*_B_ being the Boltzmann constant, *T* the absolute temperature, and *η* the viscosity of the solvent.

**Figure 3:**
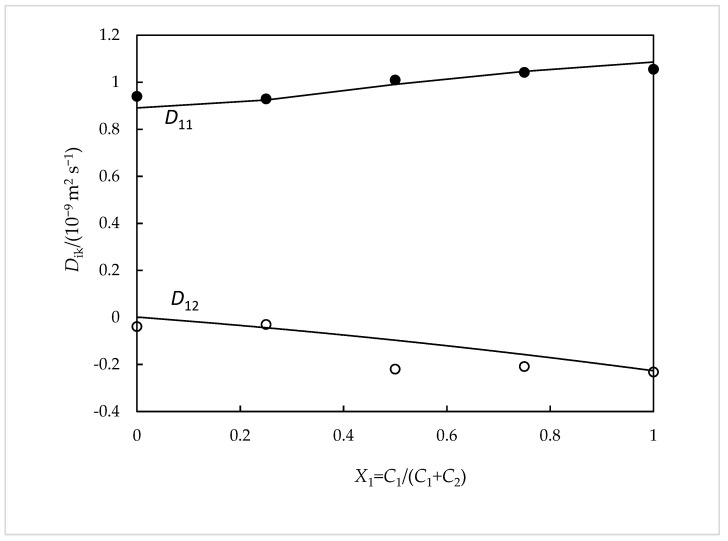
Ternary *D_ik_* coefficients for NaSal(*C*_1_) + β-CD (*C*_2_) aqueous solutions containing 0.010 mol dm^−3^ of total solute compared with the corresponding values calculated from Nernst equations: *D*_11_, filled circles; *D*_1_*_2_*, hollow circles. The solid curves correspond to the values calculated from the Nernst equations [3,6].

**Figure 4:**
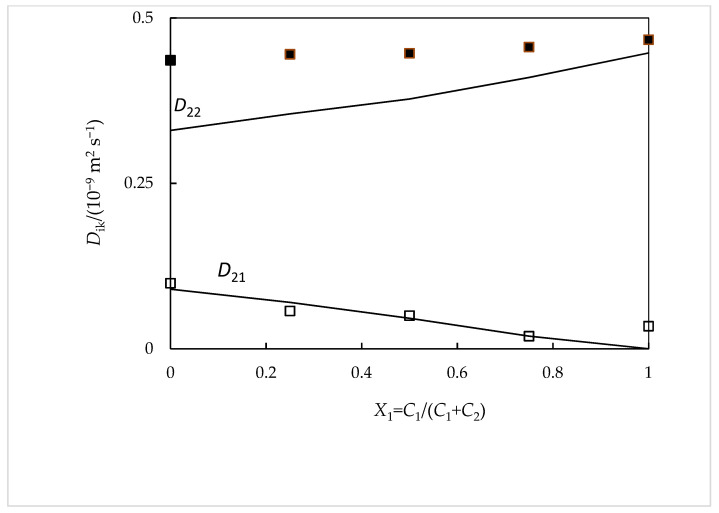
Ternary *D_ik_* coefficients for NaSal(*C*_1_) + β-CD (*C*_2_) aqueous solutions containing 0.010 mol dm^−3^ of total solute compared with the corresponding values calculates from Nernst equations: *D*_21_, hollow squares; *D*_22_, filled squares. The solid curves correspond to the values calculated from the Nernst equations [22].

From Equations 6–9 and using a value for the association constant *K*_a_ equal to 80 ± 0.1 (mol^−1^ dm^3^), the mutual diffusion coefficients *D*_11_, *D*_12_, *D*_21_, and *D*_22_ are estimated. As it can be seen in Figure 3 and Figure 4, said values thus estimated show good agreement with those measured experimentally (with deviations, in general, ≤3%). Furthermore, the value of *K*_a_ that is selected is quite close to that found in the literature (*K*_a_ = 105 ± 15 (mol^−1^ dm^3^)) [9].

### 2.3. Complex Formation

In order to evaluate the complex formation and its stoichiometry, the Job method was applied; the results obtained for both the βCD-NaSal and Na_4_EtRA-NaSal systems are shown in Figures S1 and S2 in Supplementary Materials. The Job plot for both systems shows a maximum at a mole fraction of X ~0.5, indicating that the complex formation for both systems occurs with a 1:1 stoichiometry. This fact is also in agreement with the considerations assumed for the association constant calculation.

**Figure 5:**
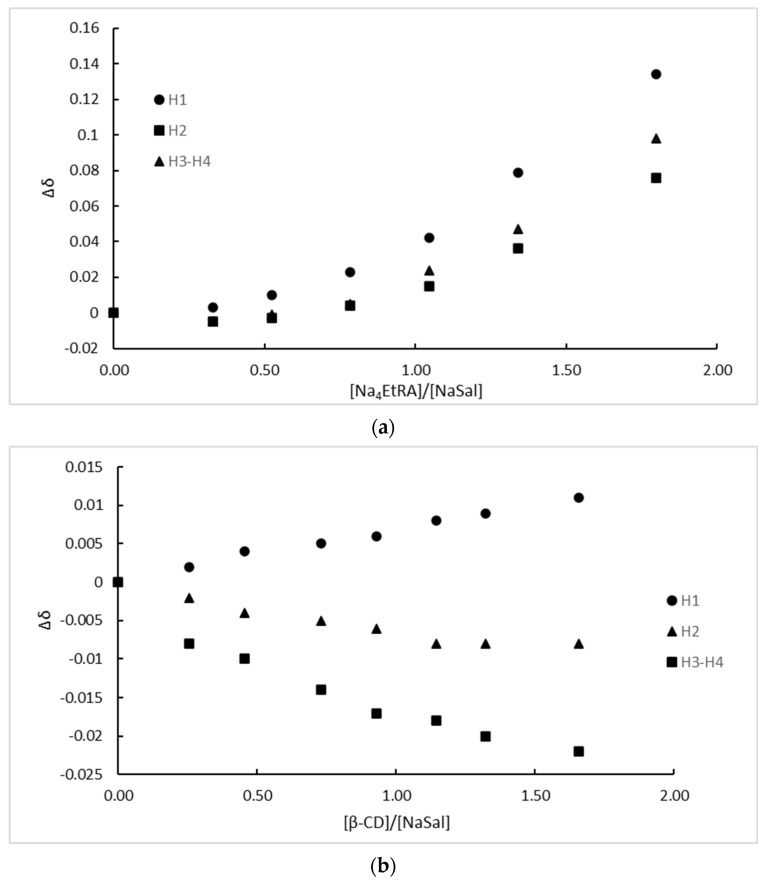
Chemical shifts as a function of the molar rate for (**a**) Na_4_EtRA-NaSal and (**b**) β-CD-NaSal.

Figure 5 shows the chemical shifts (Δδ = δ_free NaSal_ − δ_complex_.) of all NaSal protons as a function of the molar rate obtained from the NMR titration. For the Na_4_EtRA-NaSal complex, all values of Δδ increase with the molar rate (Figure 5a), presumably because of the electrostatic repulsion between the free fragments; note that both Na_4_EtRA and NaSal are anionic. In addition, all the NaSal protons are shifted considerably, which can be explained either by considering a total entry of the drug into the cavity, or by the formation of an outer-side complex. We will return to this in the computational Results section.

On the other hand, Δδ presented negative values for H_2_, H_3_, and H_4_ NaSal protons (Figure 5b), which is consistent with a total entry of the drug into the β-CD cavity, because of the electronic protection that is given in the total inclusion process. A total entry of the drug into the β-CD cavity is also consistent with the fact that all NaSal protons were perturbated.

### 2.4. Computational Studies

**Figure 6:**
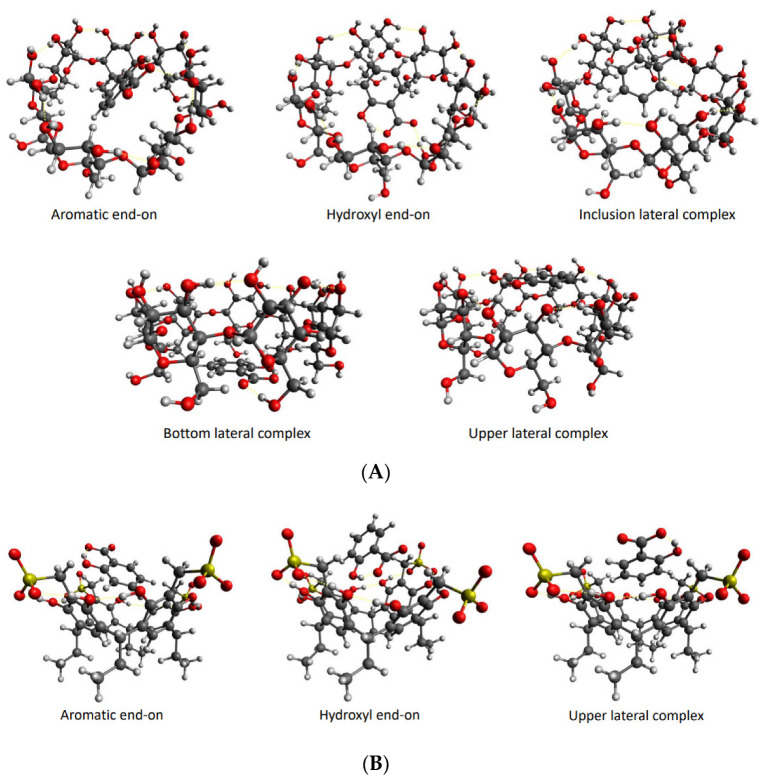
Structure of the different HG complexes obtained in the geometry optimizations. (**A**) β-CD-NaSal complex. (**B**) Na_4_EtRA-NaSal system.

Geometry optimization calculations were performed on each of the isolated carriers β-CD and Na_4_EtRA, with the NaSal, and the HG complexes to evaluate the complexation stability. For the HG complexes, different orientations of the NaSal guest in the cavity of β-CD and Na_4_EtRA were studied in such a way that both inclusion and outer-side complexes were considered. Because of the polar groups on the carriers, five different orientations of NaSal were evaluated for β-CD, whereas only three were evaluated for Na_4_EtRA. The optimized structures of these HG systems are depicted in Figure 6. Complexation energies (∆*E_C_*) were calculated according to Equation (12) for all the orientations; the values obtained are listed in Table 4, Tables S1 and S2.

(12) $$∆E_{C}=E_{complex}-E_{Carrier}-E_{Guest}$$

For the β-CD-NaSal system, the values of ∆*E_C_* show a higher stabilization for the inclusion complexes, likely because the amount of polar interactions formed inside the cavity of β-CD is higher compared to the outer-side complexes. The inclusion of NaSal inside the cavity of β-CD agrees with the deviation in the chemical shift observed in the NMR titration with respect to the isolated species. Among the different orientations, the hydroxyl side-on complex is the most stable (∆*E_C_* = −36.255 kcal mol^−1^), since it is the one that involves a greater amount of hydrogen bonds between NaSal and the hydroxyl groups of β-CD. On the other hand, the complexation energies for the Na_4_EtRA-NaSal system show a greater stability for the outer-side complex (∆*E_C_* = −20.362 kcal mol^−1^). In this system, both the guest and the host are charged species, therefore electron repulsions are not amenable with the inclusion of NaSal. Despite hydrogen bonds are not observed in the outer-side complex, long-range interactions allow their formation. Such outer lateral position of the NaSal, in the upper rim of Na_4_EtRA, is in line with the experimental results, thus the effect of the electric field generated by a concentration gradient of Na_4_EtRA is predominant, driving a coupled flow of Sal^−^ ions in the same direction as the flux of EtRA^4−^ ions, and *D*_12_ > 0. In summary, we found that the hydroxyl end-on and the upper outer-side complexes are the dominant conformations of the β-CD-NaSal and Na_4_EtRA-NaSal systems, respectively.

We also obtained the thermodynamic properties of the complexation process (∆*G_C_*, ∆*S_C_*, ∆*H_C_*) through analytical frequency calculations on the most stable complex of the β-CD-NaSal and Na_4_EtRA-NaSal systems (Table 4). ∆*G_C_* is negative for both systems; nevertheless, the β-CD-NaSal complex has the most negative value, which shows that the complexation of NaSal in β-CD is thermodynamically more favored. In addition, the formation of both complexes is exothermic (the values of ∆*H_C_* are −33.9 and −18.5 kcal mol^−1^ for the β-CD-NaSal and Na_4_EtRA-NaSal, respectively) indicating the stabilization by long-range interactions [23]. Furthermore, the higher value of ∆*H_C_* for Na_4_EtRA-NaSal can be associated with higher repulsion due to host–guest interactions.

We also calculated the solvation free energies (∆*G_solv_*) of the complexes (Table 4). The more negative value found for the Na_4_EtRA-NaSal complex (∆*G_solv_* = −646.8 kcal mol^−1^) is consistent with the more favorable interactions between the HG complex and the solvent. In the Na_4_EtRA-NaSal complex, the polar groups of NaSal are oriented out of the cavity of the carrier, whereas in the β-CD-NaSal system, the complete inclusion that occurs reduces the solvent-NaSal interactions. These observations can explain the negative values of *D*_12_ for β-CD-NaSal. Most of the free salicylate is consumed by the formation of β-CD-NaSal inclusion complexes. Consequently, NaSal diffuses toward the region of higher β-CD concentration, resulting in counter-current flow to the main β-CD.

**Figure 7:**
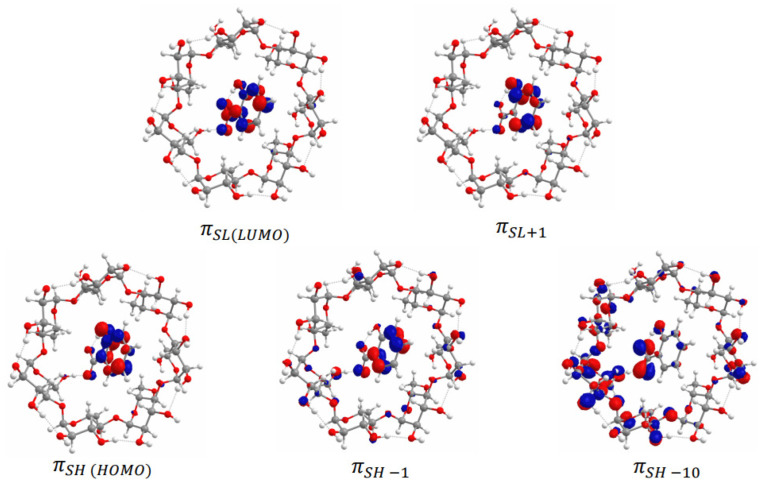
Molecular orbitals of the β-CD-NaSal complex.

**Figure 8:**
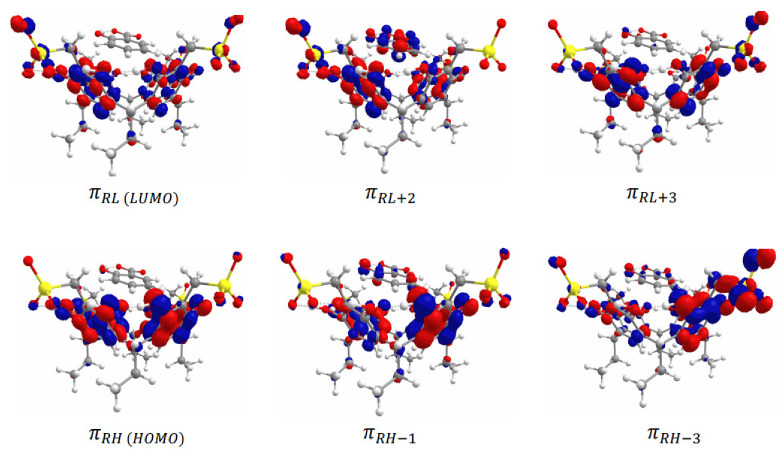
Molecular orbitals of the Na_4_EtRA-NaSal complex.

The absorption spectra of the β-CD-NaSal and Na_4_EtRA-NaSal complexes have also been simulated by TD-DFT calculations (Figures S3 and S4, Supplementary Materials). Initially, the electronic structure of the ground state of both complexes have been analyzed in terms of their frontier molecular orbitals (Figure 7 and Figure 8). The HOMO and LUMO of the β-CD-NaSal complex are localized on the aromatic ring of the NaSal moiety. These orbitals have a π nature, which encourages the formation of the inclusion complex (Figure 7). It is worth mentioning that in both complexes, the orbitals are strongly located on each fragment, supporting the absence of covalent interactions. Regarding the Na_4_EtRA-NaSal complex, the HOMO and LUMO are localized mainly in the RS fraction. In this complex, NaSal is stabilized by π-π stacking with the resorcinol rings of Na_4_EtRA in a lateral placement (Figure 8). Concerning the absorption wavelengths, a good agreement is obtained with the bands observed in the experimental spectra, Table 5. The band at 256 nm of β-CD-NaSal arises from the population of the A^1^ state, that band is associated to an intramolecular π-π transition in the NaSal fragment. The two lowest energy bands at 210 and 182 nm result from the population of the 4 ^1^A and 5 ^1^A states, respectively. These states are also mainly the result of π-π electronic transitions in the NaSal fragment.

The bands observed in the absorption spectrum of Na_4_EtRA-NaSal at 252 and 213 are associated to the 3 ^1^A and 13 ^1^A states, respectively. These two states arise from π-π electronic transitions between the resorcinol rings of Na_4_EtRA. Nevertheless, the 3 ^1^A state presented a non-negligible CT transition from NaSal to Na_4_EtRA. Furthermore, the band at 213 also presented a contribution of the 16 ^1^A state, which is related to another CT transition between the HOMO orbital of Na_4_EtRA and the $\pi_{SL+2}$ orbital of NaSal. The simulated UV–vis spectra of both systems, Na_4_EtRA-NaSal and β-CD-NaSal, are in line with those obtained experimentally. This fact supports the formation of the complexes described in this study.

## 3. Materials and Methods

### 3.1. Materials

Table 6 lists the chemicals used in this work: sodium salicylate (Panreac, mass fraction purity > 0.99; molar mass = 160.11 g mol^−1^), β-cyclodextrin (mass fraction purity > 0.97; molar mass = 1134.98 g mol^−1^), and tetrasodium 5,11,17,23-tetrakissulfonatemethylene-2,8,14,20-tetra(ethyl)resorcin[4]arene (Na_4_EtRA). This last compound was synthesized according to what was reported in the literature [24]. All these chemicals were used without further purification, but they were stored under low pressure in a desiccator over silica gel.

All solutions were freshly prepared by using Millipore Milli-Q water (specific resistance = 18.2 MΩ cm, at 298.15 K). For diffusion measurements, these were prepared (at 25 °C) in calibrated volumetric flasks. For the other measurements, they were prepared by the direct weighing of both solute and solvent. To determine its concentration, the water content of the corresponding chemicals was taken into account. Before each experiment, solutions were de-aerated for 30 min, approximately.

### 3.2. Ternary Diffusion by Taylor’s Method: Concepts and Some Experimental Aspects

For a ternary system {component (1) + component (2) + water}, the diffusion flux of component *i* in the direction of its concentration gradient, *J_i_*, can be determined by Fick’s equations [25]:

(13) $$J_{i}=-D_{ii}\nabla C_{i}-D_{ik}\nabla C_{k}$$

being *D_ii_* (*i*: 1 or 2) the main ternary mutual diffusion coefficients (representing the flow of component *i* caused by its own concentration gradient) and *D_ik_* the cross-diffusion coefficients (representing the coupled flow of component *i* caused by the gradient of the other component, *k*).

One of the most extensively used experimental techniques to determine values of these diffusion coefficients is the Taylor dispersion technique, which is very well described in the literature [12,26,27,28]. In this work, each solution studied flowed in a laminar way (constant flow rate of 0.17 mL min^−1^ with retention times, *t*_R_ ≈ 1.1 × 10^4^ s, by using a Gilson Minipuls-3 metering pump) through a long capillary tube (length 3048.0 (±0.1) cm, and internal radius 0.03220 (±0.00003) cm) which was kept inside an air thermostat at 298.15 K. For given times, small amounts (0.063 mL) of a solution with a composition slightly different from that of the solution under study were successively injected into said capillary tube (with the help of a Rheodyne valve, model 5020) and its dispersion was precisely monitored, at 5 s intervals, with the help of both a Waters 2410 digital refractometer and an Agilent 34401 A digital voltmeter was placed at the end of the aforementioned capillary tube.

The voltages measured as a function of the elution time, *V*(*t*), define peaks whose shape responds to a Gaussian distribution and whose height depends on the difference in concentration between the solution under study and the injected one. The values corresponding to at least two pairs of these peaks, for each solution studied, were fitted to Equation (14):

(14) $$V\left(t\right)=V_{0}+V_{1}+V_{max}{\left(t_{R}/t\right)}^{1/2}\left[W_{1}exp\left(-\frac{12D_{1}{\left(t-t_{R}\right)}^{2}}{r^{2}t}\right)+\left(1-W_{1}\right)\;exp\left(-\frac{12D_{2}{\left(t-t_{R}\right)}^{2}}{r^{2}}\right)\right]$$

to obtain the values of the different *D_ik_* coefficients. In equation (14) *D_i_* (*i*: 1 or 2) represents the eigenvalues of the matrix of the ternary *D_ik_* coefficients; *V*_0_, *V*_1_ and *V_max_* are the baseline voltage, the baseline slope and the peak high, respectively; and *W*_1_ and (1 − *W*_1_) are normalized pre-exponential factors.

### 3.3. Complex Formation

In order to study the complex formation and its stoichiometry, the Job method was performed. The experiments were carried out using a Shimadzu UV-2450 spectrophotometer. The Job method was performed by adding an aqueous host solution to an aqueous guest solution whose concentration varied from 0 to 1.0 mol fraction. The absorbance of the solutions was measured at a wavelength of *λ_max_* = 286 nm for the NaSal-Na_4_EtRA system and *λ_max_* = 296 nm for the NaSal-β-CD.

An NMR titration was performed at 298.15 K using a Varian of 400 MHz spectrometer with a 5 mm probe. Residual solvent (HOD) at pre-saturation was used to perform the spectra; 24 k data points covering a spectral width of 8 kHz were obtained, the radiofrequency excitation pulse was 45° and the scan repetition time was 15 s to allow for complete relaxation. The resonance of the Si-(CH3)3 signal in TSP was used as internal reference, assigning it a shift value of 0 ppm. Samples were prepared in D_2_O (Eurisotop, 99.9%) as solvent, and tracer amounts of 3-(trimethylsilyl)propionic-2,2,3,3-d4 acid sodium salt (TSP), from Sigma. The NMR titration was performed by adding a carrier aqueous solution (8mM) to sodium salicylate aqueous solution (2.9mM), during the entire titration process, no precipitate was observed [29]. The results were analyzed using the HypNMR software [13].

### 3.4. Computational Details

The computational studies for the complex formation energies and the absorption wavelengths were performed in the framework of the Density Function Theory (DFT). In all calculations, the CAM-B3LYP functional [30] was used as in previous works [23] together with the Ahlrichs def2-SVP basis set, as included in the ORCA 4.2.1 package [31,32]. The resolution of identity approximation was taken advantage of for the calculations of Coulomb integrals with the auxiliary basis sets def2-TZVP/C [33,34,35]. The Grimme dispersion correction was also included, by the D3BJ approximation in ORCA [36] as well as the solvent effects by the Conductor-like Polarizable Continuum Model (CPCM) [37]. Water was implicitly simulated as a solvent with values of dielectric constants and refractive index of 80.4 and 1.33, respectively. The threshold for energy convergence in the self-consistent field procedure was set at 1 × 10^−8^ a.u. Geometry optimization calculations were performed on the NaSal anion, the β-CD compound, the Na_4_EtRA anion, and on its corresponding HG complex. In addition, analytical frequency calculations were performed on the optimized geometries, without obtaining imaginary frequencies. The energy of the 20 low-lying singlet states was estimated by using the TD-DFT approach [38,39]. In order to simulate the absorption spectrum of the complexes, the transition wavelengths and the corresponding oscillator strengths were calculated via the electric dipole moments. A Gaussian fitting with a linewidth of 50 nm was used for plotting the absorption bands. For the depiction of geometries and orbitals, the ChemCraft visualization tools were used [22].

## 4. Conclusions

The formation of supramolecular complexes between Na_4_EtRA-NaSal and β-CD-NaSal was studied by spectroscopic and computational techniques, as well as by determining mutual diffusion coefficients. The Job plots obtained for both systems show the formation of host–guest complexes in a 1:1 ratio. For the β-CD-NaSal complex, the analysis of the diffusion coefficients and the complexation energies obtained in the computational calculations indicates that an inclusion complex occurs, while for the Na_4_EtRA-NaSal, such inclusion complex does not occur. The effect of the electric field generated by a Na_4_EtRA concentration gradient is predominant, driving a coupled flow of Sal^−^ ions in the same direction as the flux of EtRA^4−^ consistent with the formation of an outer-side complex.

The solvation free energy for the Na_4_EtRA-NaSal complex has more negative values compared to those obtained for the β-CD-NaSal complex. In the Na_4_EtRA-NaSal complex, the polar groups of NaSal are oriented away the cavity of the carrier, whereas in the β-CD-NaSal system, total inclusion reduces the solvent-NaSal interactions. This is in line with the experimental results obtained for the diffusion coefficients, and also explains the behavior of the mutual diffusion coefficients.

Finally, the simulated UV–vis spectra of both systems agree with those obtained experimentally, which supports the formation and structure of the complexes studied.

## Figures and Tables

**Table 1:** Experimental ternary mutual diffusion coefficients, *D*_11_, *D*_12_, *D*_21_, and *D*_22_, of aqueous NaSal (*C*_1_) + Na_4_EtRA (*C*_2_) solutions at *T* = 298.15 K and *P* = 101.3 kPa.

*C*_1_^a^	*C*_2_^a^	*X*_1_^b^	*D*_11_ ± *S*_D_^c^	*D*_12_ ± *S*_D_^c^	*D*_21_ ± *S*_D_^c^	*D*_22_ ± *S*_D_^c^
0.0000	0.0100	0.000	0.979 ± 0.010	0.047 ± 0.020	0.099 ± 0.025	0.711 ± 0.016
0.0025	0.0075	0.250	1.001 ± 0.015	0.060 ± 0.020	0.070 ± 0.015	0.700 ± 0.005
0.0005	0.0005	0.500	1.034 ± 0.010	0.076 ± 0.025	0.055 ± 0.012	0.660 ± 0.010
0.0075	0.0025	0.750	1.067 ± 0.011	0.410 ± 0.020	0.044 ± 0.009	0.599 ± 0.008
0.0100	0.0000	1.000	1.096 ± 0.025	1.201 ± 0.046	0.034 ± 0.020	0.499 ± 0.020

^a^*C*_1_ and *C*_2_ in units of mol dm^−3^; ^b^ *X*_1_ is the molar faction of NaSal in these solutions; ^c^ diffusion coefficients and the respective standard deviations of the mean in units of 10^−9^ m^2^ s^−1^.

**Table 2:** Experimental ternary mutual diffusion coefficients, *D*_11_, *D*_12_, *D*_21_, and *D*_22_, of aqueous NaSal(*C*_1_) + β-CD (*C*_2_) solutions at *T* = 298.15 K and *P* = 101.3 kPa.

*C*_1_^a^	*C*_2_^a^	*X*_1_^b^	*D*_11_ ± *S*_D_^c^	*D*_12_ ± *S*_D_^c^	*D*_21_ ± *S*_D_^c^	*D*_22_ ± *S*_D_^c^
0.000	0.008	0.000	0.940 ± 0.005	−0.039 ± 0.035	0.099 ± 0.020	0.436 ± 0.010
0.0025	0.0075	0.750	0.929 ± 0.007	−0.030 ± 0.030	0.057 ± 0.010	0.443 ± 0.006
0.005	0.005	0.500	1.009 ± 0.006	−0.220 ± 0.040	0.050 ± 0.025	0.446 ± 0.009
0.0075	0.0025	0.000	1.042 ± 0.003	−0.209 ± 0.030	0.019 ± 0.010	0.456 ± 0.010
0.0100	0.000	1.000	1.055 ± 0.002	−0.232 ± 0.020	0.034 ± 0.010	0.467 ± 0.009

^a^*C*_1_ and *C*_2_ in units of mol dm^−3^; ^b^ *X*_1_ is the molar faction of NaSal in these solutions; ^c^ diffusion coefficients and the respective standard deviations of the mean in units of 10^−9^ m^2^ s^−1^.

**Table 3:** Limiting diffusion coefficients, *D_s_*, of different species in the solution at *T* = 298.15 K and *P* = 101.3 kPa.

Species	*D_s_*/(10^−9^ m^2^ s^−1^)
Salicylate ion (Sal^−^)	0.918
Β-CD	0.436
Sal-β-CD	0.421

**Table 4:** Energy variation and thermodynamic properties of the complexation process.

Properties/System	Δ*E* (kcal/mol)	Δ*G* (kcal/mol)	Δ*H* (kcal/mol)	Δ*S* (kcal/kmol)	Δ*G_solv_* (kcal/mol)
β-CD-NaSal	−36.255	−17.722	−33.894	−0.0542	−94.832
Na_4_EtRA-NaSal	−20.362	−4.172	−18.485	−0.0480	−646.781

**Table 5:** Assignment of the bands observed in the absorbance spectra of the host–guest complexes.

β-CD-NaSal Complex					
State	*λ_exp_*/(nm)	*λ_cal_*/(nm)	Oscillator Strength	Dominant Electronic Transitions (%)	Type
1 ^1^A	296	256	0.156	$\pi_{SH-1}$→ $\pi_{SL+1}$ (10%) $\pi_{SH}$→ $\pi_{SL}$ (85%)	Intra NaSal Intra NaSal
4 ^1^A	230	210	0.047	$\pi_{SH-1}$→ $\pi_{SL+1}$ (46%) $\pi_{SH}$→ $\pi_{SL+1}$ (42%)	Intra NaSal Intra NaSal
5 ^1^A	202	182	0.474	$\pi_{SH-1}$→ $\pi_{SL+1}$ (35%) $\pi_{SH-1}$→ $\pi_{SL}$ (85%)	Intra NaSal Intra NaSal
**Na_4_EtRA-NaSal Complex**					
**State**	***λ_exp_*/(nm)**	***λ_cal_*/(nm)**	**Oscillator Strength**	**Dominant Electronic** **Transitions (%)**	**Type**
3 ^1^A	286	252	0.212	$\pi_{SH-1}$→ $\pi_{RL}$ (24%) $\pi_{RH}$→ $\pi_{RL+3}$ (14%)	CT NaSal-EtRA Intra EtRA
13 ^1^A	213	212	0.043	$\pi_{RH-3}$→ $\pi_{RL}$ (46%)	Intra EtRA
16 ^1^A	213	212	0.041	$\pi_{RH}$→ $\pi_{SL+2}$ (46%)	CT EtRA-NaSal

**Table 6:** Sample description.

Chemical Name	Source	CAS Number	Mass Fraction Purity	Analysis Method
Na_4_EtRA	Synthesized	--	>0.99	^1^H NMR; HPLC-qToF-PDA
Sodium salicylate	Panreac	54-21-7	>0.99	
β-Cyclodextrin	Sigma-Aldrich (Water mass fraction 0.131) ^a^	7585-39-9	>0.97	
H_2_O	Millipore-Q water (ρ = 1.82 × 10^5^ Ω m at 298.15 K)	7732-18-5		
D_2_O	Sigma-Aldrich	7789-20-0		

^a^ The mass fraction purity is on water-free basis; these data are provided by the suppliers.

## Data Availability

Data are contained within the article and Supplementary Materials.

## Supplementary Materials

Supplementary materials can be found at: https://www.mdpi.com/article/10.3390/ijms24043921/s1.
