# Supplementary material for: Interactions of Sodium Salicylate with β-Cyclodextrin and an Anionic Resorcin[4]arene: Mutual Diffusion Coefficients and Computational Study
Source: Int J Mol Sci. 2023 Feb 15;24(4):3921. doi: 10.3390/ijms24043921 (PMC9961392; doi:10.3390/ijms24043921)
Supplement: Supplementary file 1 [file ijms-24-03921-s001.zip › ijms-2195707-supplementary.pdf]

Supplementary Materials

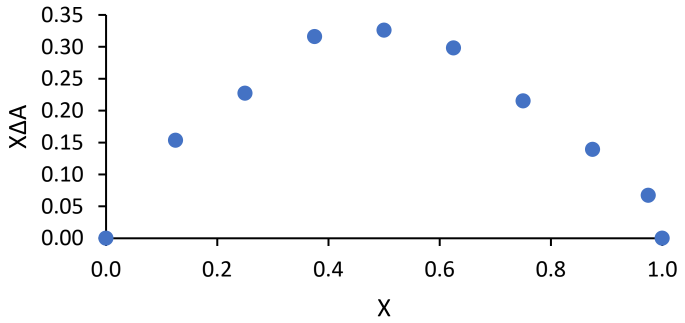

Figure S1. Job plot obtained for the system β−CD−NaSal.

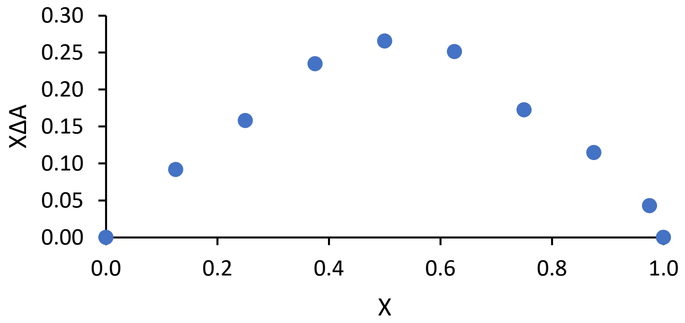

Figure S2. Job plot obtained for the system Na_4EtRA−NaSal.

Table S1. Energy variation of different β−CD−NaSal complexes.

Complex/Property	Hydroxyl end-on	Aromatic end-on	Inclusion outer-side complex	Upper outer-side complex	Bottom outer-side complex
ΔE_C (kcal/mol)	−36.255	−33.487	−30.119	−26.518	−31.984

Table S2. Energy variation of different Na_4EtRA−NaSal complexes.

Complex/Property	Hydroxyl end-on	Aromatic end-on	Upper outer-side complex
ΔE_C (kcal/mol)	−17.5290	−18.0918	−20.362

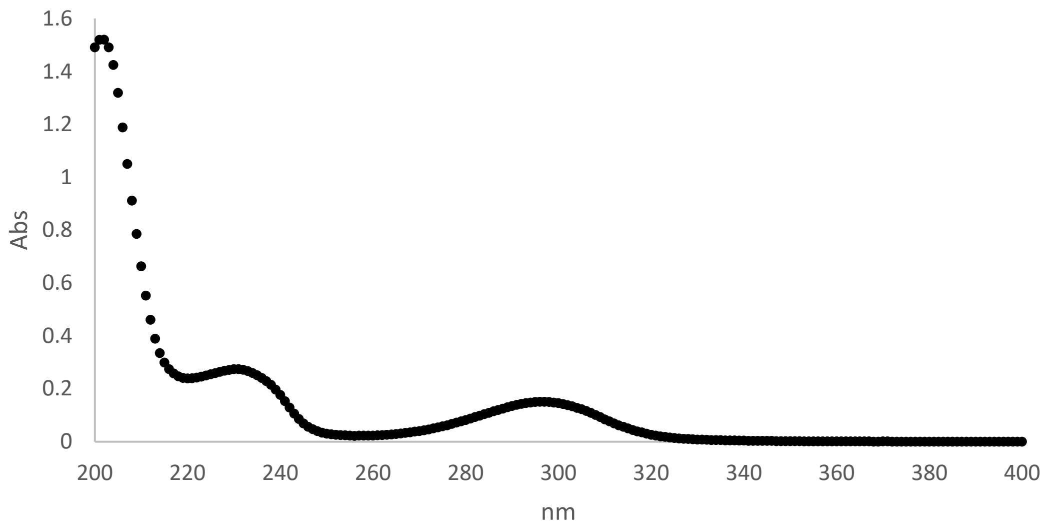

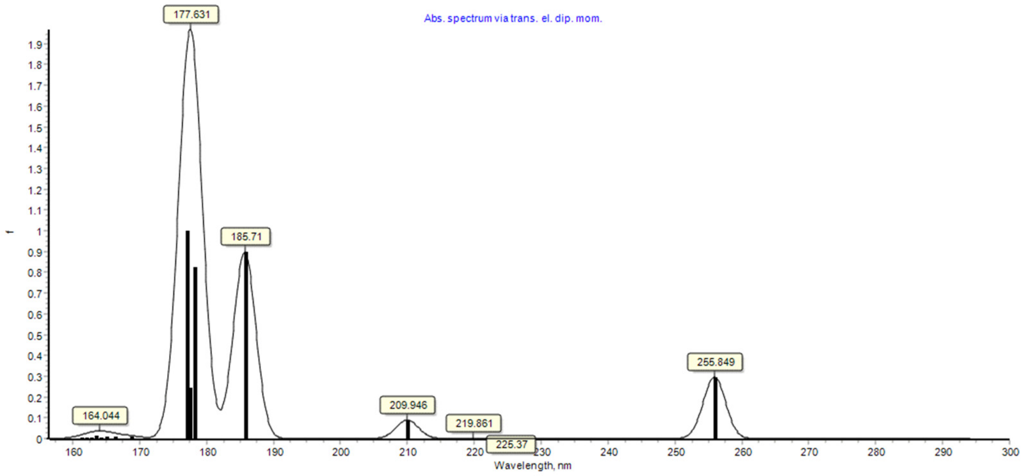

Figure S3. Upper: Experimental absorption spectrum of B−CD−NaSal complex. Bottom: Simulated absorption spectrum of B−CD−NaSal complex.

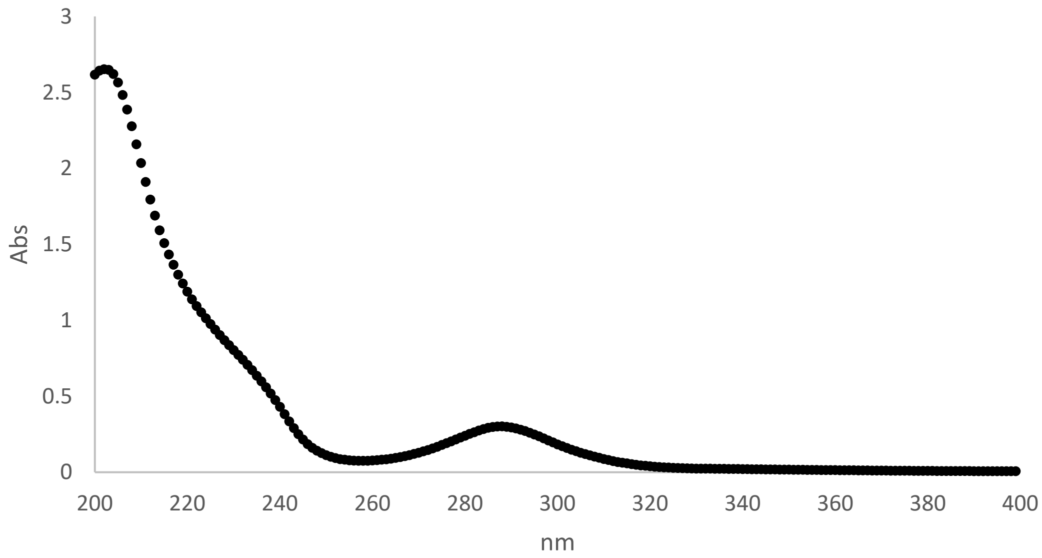

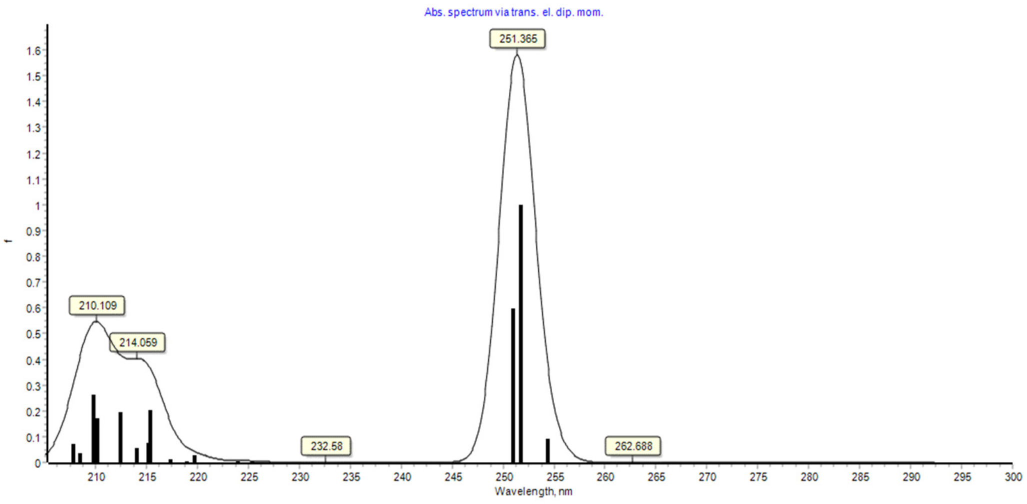

Figure S4. Upper: Experimental absorption spectrum of Na_4EtSN−NaSal complex. Bottom: Simulated absorption spectrum of Na_4EtSN−NaSal complex.
